# Health Environment and Sustainable Development

**DOI:** 10.3390/ijerph19138175

**Published:** 2022-07-04

**Authors:** Pilar Aparicio-Martínez, María Pilar Martinez-Jimenez, Alberto-Jesús Perea-Moreno

**Affiliations:** 1 Grupo Investigación Epidemiológica en Atención Primaria (GC-12), Maimonides Institute of Biomedical Research of Cordoba (IMIBIC), Department of Nursing, Pharmacology and Physiotherapy, Faculty of Medicine and Nursing, Menedez Pidal, University of Cordoba, 14071 Cordoba, Spain; n32apmap@uco.es; 2 Departamento de Física Aplicada, Radiología y Medicina Física, Campus de Rabanales, Universidad de Córdoba, 14071 Cordoba, Spain

Although there are multiple definitions of sustainability, it can be defined as the balance of a species and the resources of its environment [1]. Globally, the diverse definitions of the term embedded the concept of improving and maintaining an individual’s well-being in the long term by recovering, adjusting, or preserving environmental systems [2,3]. Sustainability is based on simultaneous benefits regarding economic, social, and environmental factors. Among all the factors contributing to sustainability, innovational and technological advances have become the most relevant elements defining a population’s health [4].

These elements connected to sustainability have been described in different reports and recommendations, such as the Brundtland Report [5,6]. This report is the beginning of the Sustainable Development Goals (SDGs), establishing an agenda for 2030 to minimize emissions and reduce climate change. This agenda includes 17 SDGs with 169 targets, with a high interest in sustainability, including health systems, human health, the social environment, social equality, and education in sustainability. Some goals integrate the environment and health, such as Goal 3, known as “Good Health and Well-being”, and those related to the environment (Goal 13 focused on Climate Action, Goal 14: “Underwater: conservation of marine fauna”, and Goal 15: “Life on land: conservation of terrestrial ecosystems”) since they directly affect people’s health and quality of life. Moreover, one goal is integrated transversal to achieve other goals, with this being Goal 4, “Quality Education”. This goal integrates the necessity of achieving sustainable education, which is related to Goal 1, “No Poverty”, and education on sustainability that bases on creating environmental awareness. Furthermore, as with Goal 6: “Ensure access to water and sanitation for all” or Goal 7: “Ensure access to affordable, reliable, sustainable, and modern energy”, the idea is that these goals are integrated with each other making them more inclusive [7,8].

## 1. Integration of Developments Sustainable Goals: Technology, Education, and Health

The United Nations indicated that to achieve these SDGs is necessary to incorporate new technologies and innovative tools in all fields [9,10]. Most of the innovations focused on integrating such technologies in the process to achieve the ideal of sustainability. The institutionalization inside the structure of diverse systems implies the integration of diverse technologies in making decisions, protocols, and policies that ultimately modify health care quality, from the diagnosis to the organization of care [11,12,13].

Besides, there are other areas that have integrated sustainability in their structural pillar, from creation and design up to marketing, such as the automotive industry, resulting in the concept of industry 4.0. The objective of improving all these different areas to achieve individual wellbeing is sometimes lost within technological advances since some of these technologies also contribute to health problems, from musculoskeletal issues to mental health [14,15]. Simultaneously, several economic, social, or organizational interests utilize the novel advances despite the sustainability and, therefore, the population’s well-being [16,17].

Based on this, two concepts have arisen during the last two decades, the sustainability of healthcare innovations and social sustainability. Both concepts are based on the adequation of the technologies; first, to the environment and resources and, later, to adapt them to the community [18,19,20].

Meanwhile, via the endorsements of the world of the 2030 Agenda for Sustainable Development Goals, this structured agenda is aimed at making education accessible to all and creating the foundation for sustainable development and peace. In this sense, UNESCO coordinates the international community’s action to achieve education of quality, sustainable and accessible to all people regardless of gender, ethnic background, or socioeconomic status. Education is the tool for social transformation, whose fundamental role is to generate change and guide personal and collective action. The Education for Sustainable Development (ESD) is UNESCO’s education sector’s response to the urgent and dramatic challenges facing the planet, like multiresistant bacteria, water pollution and microplastics. ESD for UNESCO’s 2030 Education Agenda aims to achieve the personal and social transformation needed to change the course of the current climate change and unbalance environment [21,22].

Finally, it is also important to highlight how the concept of e-health is currently evolving in parallel with that of smart cities, which also aims to introduce the widespread use of ICTs in cities to improve the quality of life of their inhabitants, considering important concepts, such as sustainability, energy efficiency, healthy mobility, health, and social sustainability. Smart cities make extensive use of sensors of all kinds that can be used to improve the health of their citizens, giving rise to the concept of smart health [23].

**Figure 1:**
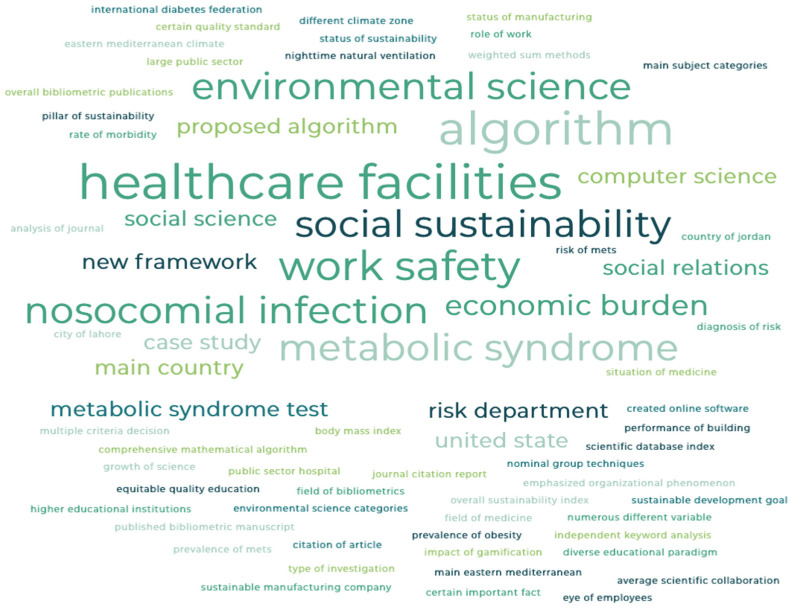
Word cloud of most relevant and frequent terms from the researchers in the Special Issue.

This Special Issue aims to advance the contribution of a Health Environment and Sustainable Development. Leading authors have published important publications in this field, with the most relevant and frequent areas analyzed being “social sustainability”, “work safety”, “environmental science “, and “healthcare facilities”, among others less common, such as “social relations” or “computer science” (Figure 1).

In this sense, Yasaman Parsia and Shahryar Sorooshian proposed a decision-making algorithm for the rearchitecting of healthcare facilities to minimize nosocomial infection risks. The algorithm was validated by implementing an HF as a case study, reaching good results.

Meanwhile, Manuel Vaquero Alvarez, Pilar Aparicio-Martinez, Francisco Javier Fonseca Pozo, Joaquín Valle Alonso, Isabel María Blancas Sánchez, and Manuel Romero-Saldaña validated the NIM-MetS test, previously used in the adult population, for the early and sustainable detection of MetS in children and adolescents. They concluded that this method shows high diagnostic accuracy, with high sensitivity, specificity, and clinic concordance with the reference test (NCEP ATP III).

Sepehr Hendiani, Huchang Liao, Morteza Bagherpour, Manuela Tvaronavičienė, Audrius Banaitis, and Jurgita Antucheviciene proposed a new benchmark approach that aims at measuring the current level of sustainability in manufacturing systems, identifying the weak points that harm the overall sustainability level and enhancing the efficiency of weak points to uplift the overall sustainability index subsequently.

Mila Cascajares, Alfredo Alcayde, Esther Salmerón-Manzano, and Francisco Manzano-Agugliaro carried out a bibliometric analysis to assess the status of medicine and environmental sciences in the scientific world through their publications. The leading countries in publications within these fields are China, USA, and Spain.

Zia Ullah, Mohammed Ali Bait Ali Sulaiman, Syed Babar Ali, Naveed Ahmad, Miklas Scholz, and Heesup Han studied the importance of occupational safety in improving social sustainability in public hospitals. Within this study, data were collected from 431 healthcare professionals in a public hospital in the city of Lahore, Pakistan and analyzed using structural equation modeling.

Jihan Muhaidat, Aiman Albatayneh, Mohammed N. Assaf, Adel Juaidi, Ramez Abdallah, and Francisco Manzano-Agugliaro analyzed new strategies to reduce the energy demand associated with cooling in residential buildings in different climates. For this purpose, the authors proposed movable window shading and night ventilation.

Johanna Andrea Navarro-Espinosa, Manuel Vaquero-Abellán, Alberto-Jesús Perea-Moreno, Gerardo Pedrós-Pérez, Maria del Pilar Martínez-Jiménez, and Pilar Aparicio-Martínez analyzed the positive and negative effects that can be derived from the use of gamification in higher educational institutions. In this study, the authors argue that gamification produces motivation and performance improvement among students and is a fundamental tool for creating sustainable higher educational institutions.

Yingyi Zhang evaluated a parametric form-based code for the sustainable development of urban communities. For this purpose, leadership methods in energy and environmental design for neighborhood development were used.

Mihaela-Roberta Stanef-Puică, Liana Badea, George-Laurențiu Șerban-Oprescu, Anca-Teodora Șerban-Oprescu, Laurențiu-Gabriel Frâncu, and Alina Crețu analyzed scientific publications dealing with the topic of “green jobs” over the last five years in order to detect global trends and associated terms.

Based on the articles, it could be determined that this Special Issue has reflected how SDGs have been analyzed from different fields, from the industry and education to healthcare systems. Besides, an interesting topic has arisen from work published, the use of models and algorithms based on significant technologies, such as artificial intelligence, to minimize the climate change impact, improve the sustainability of systems, and the decision-making of important actors in creating a viable future.

## 2. List of Contributions

1. Parsia, Y.; Sorooshian, S. A Decision-Making Algorithm for Rearchitecting of Healthcare Facilities to Minimize Nosocomial Infections Risks. *Int. J. Environ. Res. Public Health* **2020**, *17*, 855.
2. Vaquero Alvarez, M.; Aparicio-Martinez, P.; Fonseca Pozo, F.J.; Valle Alonso, J.; Blancas Sánchez, I.M.; Romero-Saldaña, M. A Sustainable Approach to the Metabolic Syndrome in Children and Its Economic Burden. *Int. J. Environ. Res. Public Health* **2020**, *17*, 1891.
3. Hendiani, S.; Liao, H.; Bagherpour, M.; Tvaronavičienė, M.; Banaitis, A.; Antucheviciene, J. Analyzing the Status of Sustainable Development in the Manufacturing Sector Using Multi-Expert Multi-Criteria Fuzzy Decision-Making and Integrated Triple Bottom Lines. *Int. J. Environ. Res. Public Health* **2020**, *17*, 3800.
4. Cascajares, M.; Alcayde, A.; Salmerón-Manzano, E.; Manzano-Agugliaro, F. The Bibliometric Literature on Scopus and WoS: The Medicine and Environmental Sciences Categories as Case of Study. *Int. J. Environ. Res. Public Health* **2021**, *18*, 5851.
5. Ullah, Z.; Sulaiman, M.A.B.A.; Ali, S.B.; Ahmad, N.; Scholz, M.; Han, H. The Effect of Work Safety on Organizational Social Sustainability Improvement in the Healthcare Sector: The Case of a Public Sector Hospital in Pakistan. *Int. J. Environ. Res. Public Health* **2021**, *18*, 6672.
6. Muhaidat, J.; Albatayneh, A.; Assaf, M.N.; Juaidi, A.; Abdallah, R.; Manzano-Agugliaro, F. The Significance of Occupants’ Interaction with Their Environment on Reducing Cooling Loads and Dermatological Distresses in East Mediterranean Climates. *Int. J. Environ. Res. Public Health* **2021**, *18*, 8870.
7. Navarro-Espinosa, J.A.; Vaquero-Abellán, M.; Perea-Moreno, A.-J.; Pedrós-Pérez, G.; Martínez-Jiménez, M.d.P.; Aparicio-Martínez, P. Gamification as a Promoting Tool of Motivation for Creating Sustainable Higher Education Institutions. *Int. J. Environ. Res. Public Health* **2022**, *19*, 2599.
8. Zhang, Y. Evaluating Parametric Form-Based Code for Sustainable Development of Urban Communities and Neighborhoods. *Int. J. Environ. Res. Public Health* **2022**, *19*, 7983.
9. Stanef-Puică, M.-R.; Badea, L.; Șerban-Oprescu, G.-L.; Șerban-Oprescu, A.-T.; Frâncu, L.-G.; Crețu, A. Green jobs—a literature review. *Int. J. Environ. Res. Public Health* **2022**, *19*, 7998.
